# Trackways Produced by Lungfish During Terrestrial Locomotion

**DOI:** 10.1038/srep33734

**Published:** 2016-09-27

**Authors:** Peter L. Falkingham, Angela M. Horner

**Affiliations:** 1 Liverpool John Moores University, School of Natural Sciences and Psychology, Liverpool, UK; 2 Department of Biology, California State University San Bernardino, San Bernardino, California, USA

## Abstract

Some primarily aquatic vertebrates make brief forays onto land, creating traces as they do. A lack of studies on aquatic trackmakers raises the possibility that such traces may be ignored or misidentified in the fossil record. Several terrestrial Actinopterygian and Sarcopterygian species have previously been proposed as possible models for ancestral tetrapod locomotion, despite extant fishes being quite distinct from Devonian fishes, both morphologically and phylogenetically. Although locomotion has been well-studied in some of these taxa, trackway production has not. We recorded terrestrial locomotion of a 35 cm African lungfish (*Protopterus annectens*; Dipnoi: Sarcopterygii) on compliant sediment. Terrestrial movement in the lungfish is accomplished by planting the head and then pivoting the trunk. Impressions are formed where the head impacts the substrate, while the body and fins produce few traces. The head leaves a series of alternating left-right impressions, where each impact can appear as two separate semi-circular impressions created by the upper and lower jaws, bearing some similarity to fossil traces interpreted as footprints. Further studies of trackways of extant terrestrial fishes are necessary to understand the behavioural repertoire that may be represented in the fossil track record.

Terrestrial locomotion has evolved multiple times in phylogenetically disparate fishes, usually in conjunction with air-breathing^1^. Of the extant fishes that employ terrestrial locomotion, a diversity of structures are used to interact with the substrate, including whole bodies, heads, and specialised pectoral fins^1^,^2^. Additionally, benthic, appendage-driven locomotion is utilised by some fishes that are fully aquatic, such as stingrays, sharks, skates, and possibly some extinct placoderm fishes^3^,^4^,^5^,^6^,^7^,^8^. The diversity of behaviours produced by these taxa is not always well predicted by morphology.

Despite their phylogenetic and morphological differences from early tetrapodomorphs, terrestrial locomotor behaviours from extant, derived Actinopterygian fishes have been invoked as offering insight into how stem tetrapods may have moved. Although Actinopterygian fishes were uncommon in the Devonian, several modern taxa have evolved ‘fin-driven’ locomotion on land, and thus can be useful models for investigating intermediate stages between fully aquatic and terrestrial locomotion (e.g., ‘walking’ catfish^1^, ‘crutching’ mudskipper^9^). However, these taxa tend to exhibit specialisations of the pectoral appendages rather than the pelvic fins—the latter of which is considered by some to be a major tetrapod innovation^10^,^11^,^12^.

Terrestrial locomotion, particularly at water margins, will likely involve travelling over compliant substrates, and in doing so, the formation of trackways. Trace fossils are complimentary to osteological remains, and when body fossils are absent or rare tracks may provide the only record of extinct life, or substantially add to what is known from body fossils alone. Trace fossils from the Devonian are of particular interest, due to their importance in informing the locomotor modes involved in the vertebrate water-to-land transition. Fossilized trackways can provide a direct record of that locomotion, as opposed to the information garnered indirectly from biomechanical models based on osteology^13^ or hypothesised from extant analogues such as salamandroid amphibians^14^,^15^ and terrestrial fishes^9^,^16^.

Besides giving rise to tetrapods, the Devonian contained an extraordinary diversity of fishes, many of which are either now extinct (e.g., Placoderms, spiny sharks) or reduced to a few relic genera (i.e., Sarcopterygii, agnathans). Although it is impossible to know for certain how many Devonian taxa made terrestrial forays, the diversity of locomotory behaviours exhibited by extant, morphologically diverse semi-aquatic fishes^1^ suggests there was a potential for Devonian fish to produce enigmatic sub-aerial traces.

Trackways with clear digit impressions from the appropriate age rock may unequivocally be assigned to a tetrapod trackmaker, and similarly, continuous sinusoidal trackways lacking footprints may easily be assigned to an aquatic, laterally undulating trackmaker e.g. refs 17,18. However, trackways of intermediate morphologies (representing intermediate behaviours) are essentially unknown. Given the scarcity of reported trackways attributed to terrestrially locomoting aquatic organisms, it seems possible that the inability to accurately identify the trackmaker may have doomed such traces to have been ignored or misidentified. Furthermore, the continuum of benthic, near-shore, and fully terrestrial substrate interactions that many of these species engage in create a variety of impressions that, once preserved, would be equally difficult to match to environment.

Sarcopterygian fishes are now reduced to a few relic taxa, but given their closer phylogenetic position to tetrapods, have made an arguably stronger case to be models of ancestral tetrapod locomotion than other fishes. Despite the phylogenetic proximity to early tetrapods, extant lungfish like the elongate, slender-finned African lungfish *Protopterus annectens* are quite morphologically dissimilar to Sarcopterygians of the Devonian, which tended to be heavy-scaled, heavy-skulled and fully lobe-finned fishes (e.g., *Soederberghia, Griphognathus, Chirodipterus*^19^,^20^). However, *Protopterus* shares morphological similarities in overall body shape, to sub-adult Australian lungfish (*Neoceratodus forsteri*), the only extant species of lungfish with fully fleshy-lobed fins (as adults). Given that *Protopterus* and other slender-finned lungfish may be paedomorphic^21^, the potential for some extinct forms to have resembled *Protopterus* at some stage in ontogeny is intriguing. More compellingly, neuromuscular control of locomotion tends to be a highly conserved trait; thus even dissimilar-appearing organisms may have similar activation patterns^22^.

Regardless of phylogenetic relationships to tetrapodamorphs, lungfish are an intriguing example of an occasionally terrestrial fish whose movements would be unlikely to be predicted by morphology alone. *Protopterus* employs both benthic ‘finned locomotion’^11^, a behaviour that has been suggested as a precursor to fully terrestrial locomotion in tetrapods, and fully terrestrial, axially-driven locomotion^23^. When submerged, the lungfish appears to use its paired fins to provide some propulsion through contact with the substrate^11^,^24^. However, this locomotor technique differs significantly from that used by the same animal during terrestrial locomotion^23^. On land, without the aid of water to support the body, the thin, flexible fins of *Protopterus* are insufficient to provide supportive or propulsive forces^23^. Although *Protopterus* is an elongate fish with nearly equal numbers of trunk and tail vertebrae, the laterally compressed tail is also minimally involved in terrestrial locomotion, as evidenced by lack of muscle activation during terrestrial locomotion^23^. Instead, these lungfish rely on their ossified crania and trunk to propel them on land^23^, planting the head as an anchor point around which to move the body. On compliant substrates this method of locomotion would be expected to leave traces which would be difficult to interpret. Given the morphological diversity of fishes during the Devonian and the intervening 400 million years to the present day, there is a great potential that some terrestrial fish tracks have either been ignored or misidentified. In order to document the morphology of such traces and provide an example search image, we recorded trackways formed by a live lungfish locomoting over mud and sand. The kinematic details and muscle activation patterns of terrestrial locomotion in *Protopterus* has been documented previously in multiple individuals^23^. Here we present representative data from a single individual in order to explore this behaviour in the context of track formation.

## Results

**Figure 1:**
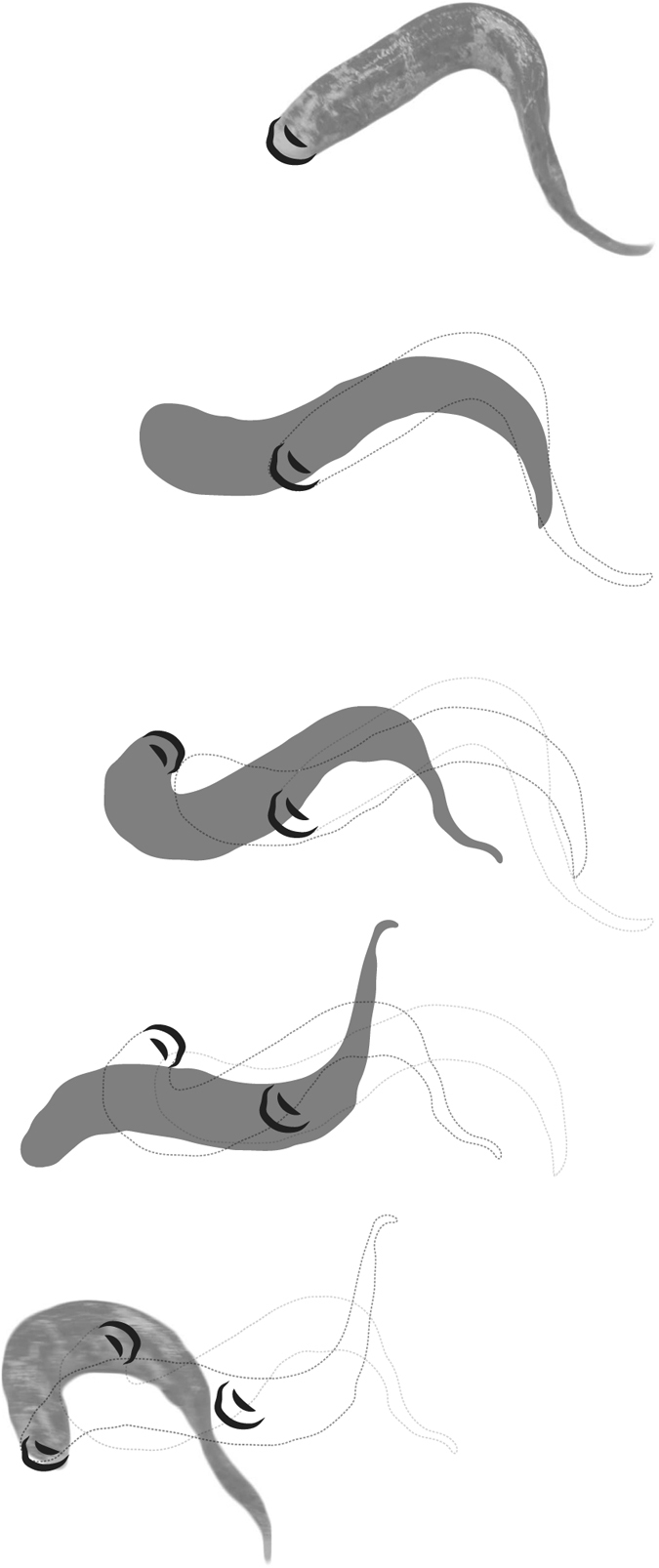
Outline images of the motion of *P. annectens* traversing soft mud. The head is planted into the sediment, and used as a pivot around which to arch the body. The process is then repeated by planting the head to the other side. Depending on the consistency of the mud, either a single impression is left, or two impressions are formed from the upper and lower mouth parts.

For each trial (total utilised trials = 10), the lungfish was placed in the centre of the substrate tray and allowed to move in any direction. Generally, the animal moved in the direction it was placed and proceeded at a rate of ~1–2 cm/s. Speed was consistently slow, even when attempts were made to elicit faster locomotion. As the lungfish traversed the compliant substrate, it did so by planting the head into the surface of the sediment, and then pivoting the rest of the body forwards (Fig. 1, Supplemental movies 1 and 2). During continuous movement, these motions occurred at a rate of ~0.25 cycles per second (that is, ~4 seconds between head plants). This ‘head-crutching’ behaviour is stereotypic and has been observed in many individuals (AMH pers. obv., 2005^23^); details of the kinematics and axial muscle activity of terrestrial locomotion in *P. annectens* are available from the literature^23^. Head plants alternated between the fish’s left and right sides between cycles, and the majority of the lungfish’s body left very little impression in the surface. The head itself, which was forced down to provide an anchor around which to pivot the body, produced a significant depression (2–9 mm deep).

**Figure 2:**
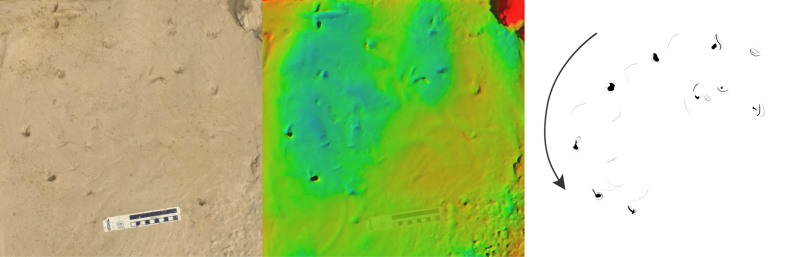
Long trail produced by *P. annectens* traversing soft mud, presented as photo (left), height mapped digital model (centre), and interpretive drawing (right). Arrow indicates direction of travel. Scale bar = 10 cm, colour height map represents 19 mm from blue (low) to red (high).

**Figure 3:**
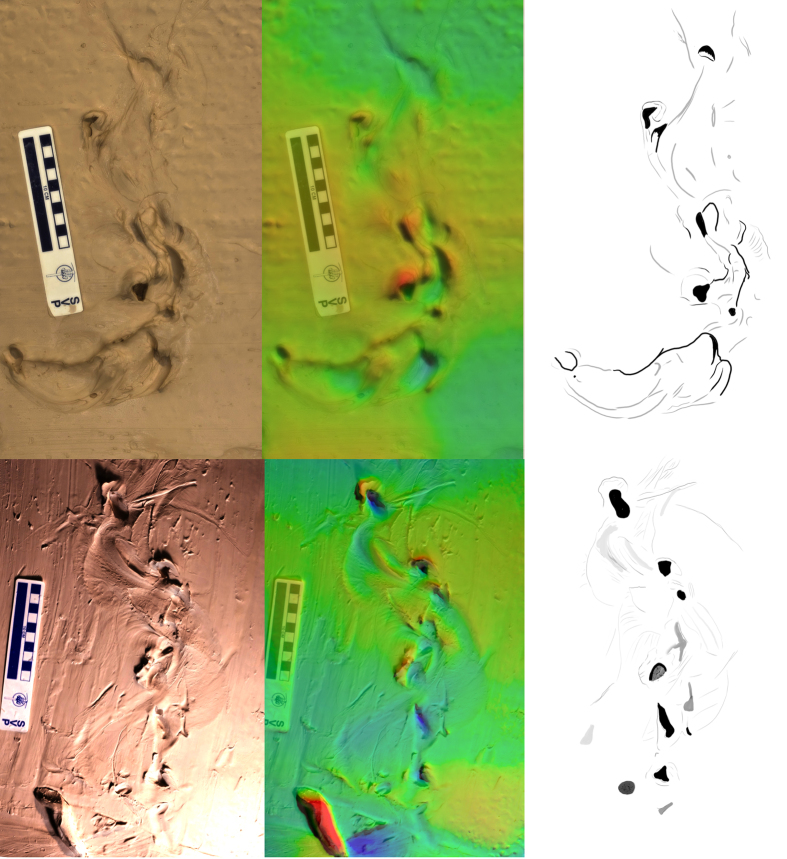
Short trails produced by *P. annactens* traversing soft mud. Marks left by the body are prominent between distinct bi-lobed impressions left by the open mouth. Direction of travel is to the top of the image, Scale bar = 10 cm. Colour map ranges from blue (low) to red (high) over 23 mm [top] and 12 mm [bottom].

**Figure 4:**
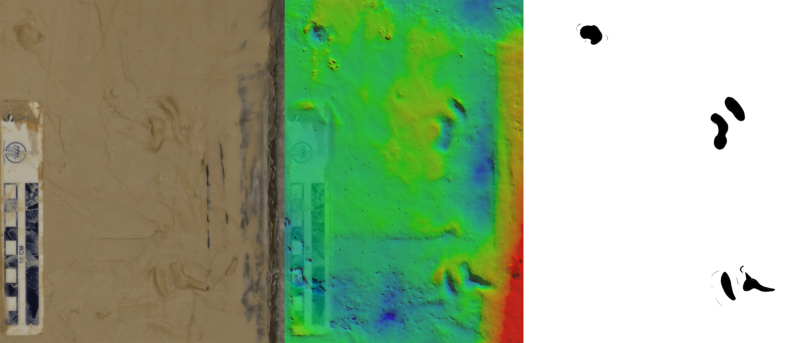
Impressions left by *P. annectens* on soft mud. The body has failed to leave any markings, while the open mouth has produced two distinct double impressions, followed by a single mark. Direction of travel is towards the upper left of the image. Scale bar = 10 cm, colour scale ranges from blue (low) to red (high) over 10 mm.

**Figure 5:**
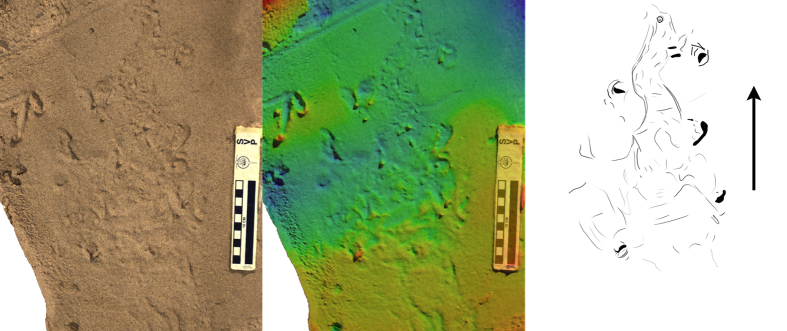
Marks left by *P. annectens* traversing moist fine sand. The body has produced a rough surface between distinct impressions left by the head being planted into the substrate. Direction of travel is towards the top of the image. Scale bar = 10 cm, colour scale ranges from blue (low) to red (high) over 15 mm.

As the lungfish moved forward, it left behind an alternating left-right sequence of head impressions (Figs 2, 3, 4 and 5). The impressions were variable in their distance from each other, (1–15 cm) but generally occurred ~10 cm apart (Figs 2, 3, 4 and 5). Between these impressions were occasional shallow sinuous markings produced by the body and fins of the animal, though such markings only substantially appeared in 2 of the trials on mud (Fig. 3), and in this case the animal was observed slipping during the pivoting manoeuvre. On sand no such markings were observed (see below). The fins were not observed to have any role in the lungfish’s locomotion. On the mud substrate, cohesion meant the pectoral fins often adhered to the trunk of the animal. Any trails left by the paired fins were a result of incidental motion against a very soft substrate.

Locomotion was generally not continuous, with the lungfish occasionally pausing for varying amounts of time (from seconds to tens of minutes). Tracks produced during continuous motion were indistinct from those produced during stop-start locomotion, which is consistent with experimental observations of birds walking on substrate, in which stopping mid-stance had no observable effect on surface track morphology (Falkingham, pers. Obs 2013). The maximum duration of continuous movement was for ~10 seconds, and in that case was terminated by reaching the edge of the sediment tray.

Head placement occurred in a semi-regular alternating sequence of left-right head plants in order to maintain locomotion in a forward direction. Occasionally the lungfish would favour one side over another with the resulting trackway veering in that direction (Fig. 2).

Often, the lungfish would impress the head into the substrate with the mouth open. In some tracks, this resulted in impressions consisting of two distinct parts, elongate or semi-circular in form (Fig. 4), created by the fleshy jaws.

On sand, the impressions left by the lungfish’s head were generally shallower (<6 mm) than on the softer mud substrate (<9 mm). The lack of cohesion meant that at the surface of the sand individual grains could be moved laterally with ease. This resulted in a highly irregular disturbed surface where the lungfish had moved (Fig. 5), rather than the distinct, more isolated impressions formed in mud (Figs 2, 3 and 4).

**Figure 6:**
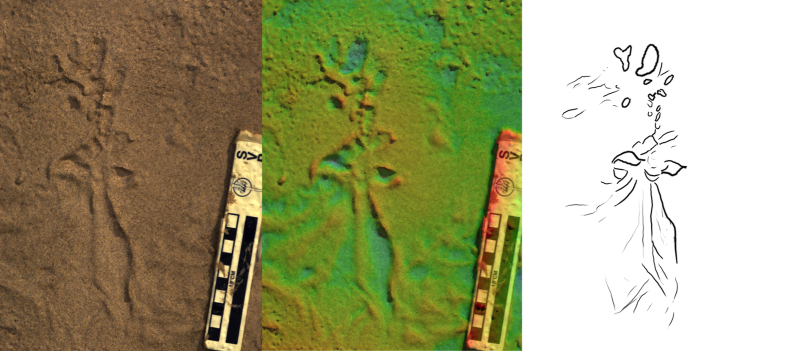
Resting trace of *P. annectens* on moist fine sand. The animal was oriented with anterior at the top of the image. Photo (left), Height map (centre), and ousupptline (right). Scale bar = 10 cm, height map covers 10 mm from low (blue) to high (red).

The lungfish was not always motivated to travel far, and occasionally declined to move at all. In one of these instances, we recorded the resting trace left behind after the animal was removed from the substrate (Fig. 6).

## Discussion

Our study shows that extant lungfish are capable of producing sub-aerial trackways consisting of rounded impressions in an alternating left-right sequence. In this regard the traces bear some resemblance to those left by limbed animals, and would not intuitively be assigned to a fish trackmaker if observed out of context.

The movement of the lungfish over a compliant substrate did not involve any significant use of the pectoral or pelvic fins. Instead, the animal propelled itself by planting the head as an anchor, and then pivoting the body forwards. This movement is powered almost entirely by axial muscles, with very little apparent contribution by the tail or fins^23^.

In the quest for finding a suitable modern analogue for tetrapod locomotion, biologists have sampled behavioural data from a wide array of extant fishes^1^,^9^,^11^,^16^,^25^,^26^,^27^,^28^,^29^, but to our knowledge few data have been collected on the trackways these fishes produce. We therefore hope that our data constitutes a preliminary search image for trace fossils left by terrestrially locomoting fishes.

### Using extant fishes as analogues for tetrapod terrestrialisation—challenges and future directions

The modern diversity of locomotory behaviours of amphibious organisms provides a broad functional landscape that may have a great deal of overlap with tetrapodomorph locomotion; convergence is common among organisms adapted for a specific habitat^30^,^31^. Lacking direct evidence of the locomotory behaviour of transitional forms, researchers investigating the vertebrate water-to-land transition must attempt to utilise modern analogues that fit within the physical realm of possibility gleaned from the sparse fossil record.

Although much emphasis is placed on appendage-driven locomotion in stem tetrapods, in extant amphibious organisms there is a tremendous diversity of both axial and appendicular structures that interact with the substrate to produce movement. The mudskipper (*Periophthalmus argentilineatus*) has been hailed as a possible analogue for early tetrapod locomotion^13^,^16^, as it relies almost entirely on its pectoral appendages to elevate and propel the body forward^16^,^25^ with some apparent contribution of tail on inclined surfaces^16^. However, the far more common strategy for terrestrial locomotion among reduced-limbed ectotherms broadly, is lateral undulation. Much of the muscle mass of fishes and salamandroid amphibians is in the axial muscles^32^,^33^, and consequently the axial structures often contribute a significant proportion of propulsive force in terrestrial locomotion, even when appendicular structures are present^1^,^23^,^34^,^35^. Stem tetrapods had similar body proportions e.g. refs 36,37 and therefore locomotion was likely to be at least *in part* driven by axial structures with variable assistance from appendicular structures (but see ref. 38).

Axially-driven locomotion need not resemble the ‘traveling’ wave sinusoidal locomotion employed by elongate ectotherms such as snakes, eels, and ropefish, however^26^,^39^,^40^. As described here and elsewhere, lungfish perform an entirely different form of terrestrial locomotion that employs a static, or ‘standing’ wave of bending^23^. This primarily axially-driven mode of locomotion is not unique to lungfish. “Walking” catfish (*Clarias batrachus*) have been documented as using a combination of tail propulsion and pectoral appendage planting to navigate terrestrially^1^,^41^. Although the catfish interact with the substrate using different anatomical structures than lungfish, the similar strategy of anchoring and arcing the body may produce grossly similar trackways to that of lungfish.

Understanding the trackways produced by extant terrestrially locomoting fishes is important not only to distinguish them from other traces in the fossil record, but also to provide a wider search context for trackways produced by tetrapodomorphs potentially using axial, rather than appendicular structures in their first forays onto land. Further investigation of lungfish and other extant terrestrially-locomoting fish trackways may offer important insight with regard to interpreting enigmatic trace fossils from the Devonian and elsewhere in the fossil record.

### Similarities with terrestrial tetrapod tracks

**Figure 7:**
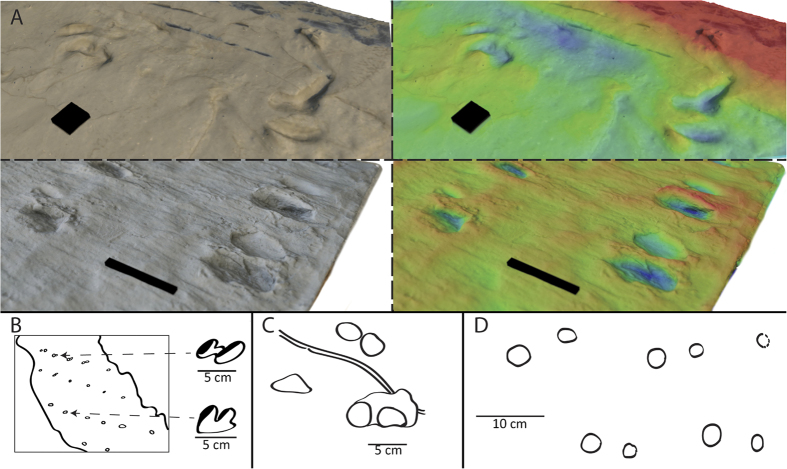
Devonian trace fossils bearing various similarities with lungfish traces; (**A**) Photo textured and height-mapped digital models of lungfish traces (top, scale bar 1 cm) and tetrapod traces from Valencia Island (bottom, scale bar 10 cm). While the traces differ substantially in size, the overall form of double impressions is highly similar morphologically. (**B**) Trace attributed to a eurypterid trackmaker by^46^ – Note the double impression is highly similar to those in Figs 4 and 7A, though orientation of the individual traces is different with respect to the trackway. (**C**) trace assigned to a tetrapod trackmaker by^58^ – The paired impressions are interpreted as manus-pes imprints. The sinuous central mark was interpreted as a coincidental horizontal burrow. (**D**) A trackway from Poland described by^49^, consisting of alternating circular, paired, impressions. While the regularity of the impressions is unlike those produced by the lungfish here, the general form of the individual tracks is not dissimilar (e.g. Figs 2 and 3).

Devonian trackways consisting of rounded or paired impressions are generally attributed to tetrapods^42^,^43^,^44^,^45^, but in some cases are interpreted as being made by other organisms, including invertebrates^46^. We present four reported trackways from the Devonian in Fig. 7, from tetrapods and invertebrates, that display (to greater or lesser degrees) morphological similarities with the lungfish traces we report. We also note a similarity with extant tetrapod traces, specifically those previously recorded from salamanders moving over certain substrates (Fig. 5 of Ref. 14).

Terrestrial tetrapod tracks from the Middle Devonian will necessarily be preserved in rocks deposited at or near the water-land boundary, as the first tetrapods to move onto the land would still be dependent on water for many aspects of their biology, such as feeding and reproduction^6^. This is also the environment one would predict terrestrial fish trackways to be preserved in, given that extant lungfish and amphibious ray-finned fish occur at the water-land interface today.

Many of the documented early tetrapod trackways are poorly defined, and lack distinct digit impressions^42^,^43^,^44^,^45^. Those that do, are often accompanied by additional poorly-defined trackways^47^,^48^,^49^. Instead of possessing specific features relatable to pedal characters in the body fossils, these trackways are ascribed to tetrapods on the basis of impressions occurring in repeated, paired, alternating or opposing patterns, based on the assumption that such patterns can only be produced by a quadrupedal tetrapod. However recent work has suggested that early tetrapods similar to *Icthyostega* lacked the rotary motions needed to have produced symmetrical gait trackways like those currently known from the Middle Devonian^13^, while other work has cast doubt on a tetrapod origin for such indisctinct marks based on comparison with modern day fish feeding traces^50^, or finprint impressions of walking cavefish^12^.

The lungfish traces described here exhibit some superficial similarities to those early tetrapod tracks that lack clearly defined digit impressions or other details. If trackways such as those presented in Fig. 2 were found in rocks of Devonian age it is conceivable that they might be misinterpreted as originating from early tetrapods. This is especially the case for those traces described herein where the mouth parts have left distinct impressions – alternating paired impressions could be construed as being individual manus and pes traces, if only by the virtue of being paired. Figure 7A provides a direct comparison between the lungfish traces described here and the Devonian tetrapod tracks reported from Valencia Island^43^. Though the lungfish traces are an order of magnitude smaller, and the tetrapod traces have been subject to tectonic deformation, there is an undeniable similarity between the final form of both traces: Both are paired, elongate impressions with similar length-width ratios (4:1).

Whilst the traces discussed here are smaller than many reported early tetrapod trackways, we note that extant lungfish can grow to over a metre in length, and extinct forms reached even greater sizes^51^. Whether such extinct forms could similarly locomote terrestrially is not known, and indeed may be unlikely given the differing morphologies between extinct forms and *Protopterus*. However, given the diversity of fish in the Devonian, as well as the potentially paedomorphic nature of *Protopterus*, we consider it possible, or even likely, that fish existed that were capable of producing similar traces through this method of locomotion; though we realise that this remains speculative until more trackway data are collected from extant taxa.

There are morphological characters that may help to differentiate between trackways produced by lungfish or other terrestrially locomoting fish and those produced by early tetrapods. The first and most obvious is the presence of digit impressions. If the tracks are of sufficient detail as to preserve digit impressions, a fish trackmaker can easily be discounted^49^. However, many early tetrapod trackways lack such detail^42^,^43^,^44^,^45^,^47^.

We observed little evidence that a mid-line impression could be produced by a lungfish moving as described here. Mid-line impressions have been described from tetrapod trackways and attributed to tail or body drag marks^47^. In cases where such trackways do not possess a mid-line impression, interpretations have been posited that the trackmaker had a very short tail or was partially supported by water^43^. However, temporal averaging difficulties in interpreting tracks, particularly in marginal environments where water levels can vary repeatedly over hours or minutes, makes it difficult to know the water depth at the time of track formation. Because the body of the lungfish only contacts the substrate between pivots, it is incapable of leaving behind a distinct central trace (Fig. 2).

Regularity of impressions may be another character with which to differentiate between terrestrial fish and tetrapod trackways. Our lungfish would often produce irregular trackways (Figs 3 and 5), with head impressions unevenly spaced both in terms of length and width along the trackway. However, during bouts of consistent locomotion, the lungfish produced trackways with quite regular spacing (Fig. 2), though in these cases we note that the trackway width was particularly narrow, and subsequently pace angulation was quite high. We would encourage future work to seek out examples of lungfish trails made in the wild and over much longer distances.

## Conclusions

Like many other air-breathing fishes, the West African lungfish makes periodic forays onto land. Despite not employing paired appendages, the sub-aerial trackways left behind are composed of alternating left-right impressions, sometimes occurring in pairs. Such traces could be conflated with tracks produced by distal limbs and, if found in fossil form, mistakenly attributed to limbed organisms, or not attributed to any track maker at all. In fact, given the diversity of near-shore fishes during the Paleozoic, it seems surprising that there are no reports of trace fossils attributed to terrestrially locomoting fish. This is likely due to a lack of comparative data from extant studies, and we hope this paper is a first step in rectifying this deficiency. We advise palaeontologists to employ caution when interpreting putative tetrapod tracks or other enigmatic traces, and furthermore suggest that more trackway data are collected from extant amphibious organisms for comparative purposes.

## Materials and Methods

For this study, we used a 35 cm West African lungfish (*Protopterus annectens*). All experiments using animals were carried out in accordance with the approved guidelines of Brown University’s Institutional Animal Care and use Committee (Protocol #1211990035). The lungfish was housed in a 125 litre tank with 12:12 light/dark cycle maintained at an average temperature of ~24 °C and fed every other day with commercial carnivorous fish pellets. To produce traces, the animal was placed on a 1 m × 1 m tray filled to a depth of ~2 cm with mud or fine sand. The mud was produced by mixing Tennessee ball clay and water at a ratio of 4:1 by volume, producing a soft, but not saturated, substrate. Before each trial, the mud surface was sprayed with a small amount of water for lubrication to avoid too much cohesion to the animal. The sand was a fine grained silica sand, and was mixed with two parts water by volume. Addition of any more water resulted in pooling at the surface, indicating the consistency used was close to saturation. The lungfish was allowed to freely move in any direction. Once the animal reached the edge of the tray it was removed and replaced into the tank.

The sediment surface was then documented using close-range photogrammetry^52^. A series of photographs (16mp, Sony Nex-6, 35 mm focal length) were taken of the surface and used to generate 3D photogrammetric models using VisualSFM^53^,^54^,^55^ and PMVS/CMVS^56^,^57^. Because of the shallow nature of many of the impressions combined with uneven sediment surfaces, 3D models were rendered using a heightmap shader in Autodesk Maya to highlight topography changes. When using wet ball clay, the surface of the sediment remained highly reflective. In order to remove the reflections (which negatively affect the photogrammetric reconstruction), dry Tennessee ball clay was lightly dusted over the traces to produce a matt, speckled surface.

## Additional Information

**How to cite this article**: Falkingham, P. L. and Horner, A. M. Trackways Produced by Lungfish During Terrestrial Locomotion. *Sci. Rep.* **6**, 33734; doi: 10.1038/srep33734 (2016).

## Supplementary Material

Supplementary Information

Supplementary Movie 1

Supplementary Movie 2
