# Supplementary material for: Trackways Produced by Lungfish During Terrestrial Locomotion
Source: Sci Rep. 2016 Sep 27;6:33734. doi: 10.1038/srep33734 (PMC5037403; doi:10.1038/srep33734)
Supplement: Supplementary Information [file srep33734-s1.pdf]

Captions for supplemental Data in:

MAKING FOOTPRINTS WITHOUT LIMBS: TRACKWAYS PRODUCED BY LUNGFISH DURING TERRESTRIAL LOCOMOTION.

Peter L. Falkingham[1,*] and Angela M. Horner[2,*]

1. Liverpool John Moores University, Department of Natural Sciences and Psychology.

2. California State University, San Bernardino, Department of Biology

Supplemental Movie 1: Lungfish locomoting on moist fine sand.

Supplemental Movie 2: Lungfish locomoting on wet mud.
